# Factors associated with viral suppression and rebound among adult HIV patients on treatment: a retrospective study in Ghana

**DOI:** 10.1186/s12981-022-00447-2

**Published:** 2022-05-25

**Authors:** Stephen Opoku, Samuel Asamoah Sakyi, Nana Kwame Ayisi-Boateng, Anthony Kwame Enimil, Ebenezer Senu, Richard Owusu Ansah, Bismark Dankwah Aning, Diana Atsieno Ojuang, Doreen Nafula Wekesa, Fatima Osman Ahmed, Chidinma B. Okeke, Ama Darkoaa Sarfo

**Affiliations:** 1 Department of Medical Diagnostics, Faculty of Allied Health Sciences, Kwame Nkrumah University of Science and Technology, Kumasi, Ghana; 2 Department of Molecular Medicine, School of Medicine and Dentistry, Kwame Nkrumah University of Science and Technology, Kumasi, Ghana; 3 Department of Medicine, Kwame Nkrumah University of Science and Technology, Kumasi, Ghana; 4 Pediatric Infectious Disease Unit, Child Health Directorate, Komfo Anokye Teaching Hospital, Kumasi, Ghana

**Keywords:** HIV/AIDS, Viral suppression, Viral rebound, Antiretroviral therapy (ART)

## Abstract

**Background:**

Viral suppression remains the most desired outcome in the management of patients with Human Immunodeficiency Virus/Acquired Immune Deficiency Syndrome (HIV/AIDS) and this can be achieved by an effective Antiretroviral Therapy (ART). However, some patients who achieve viral suppression may experience viral rebound with dire consequence. We evaluated viral suppression and rebound and their associated factors among adult patients on ART in Kumasi, Ghana.

**Methods:**

This hospital-based retrospective study was conducted at the Komfo Anokye Teaching Hospital in Ghana. We reviewed the medical records of 720 HIV patients on ART. Statistical analyses were performed using SPSS Version 26.0 and GraphPad prism version 8.0. *p* < 0.05 was considered statistically significant.

**Results:**

Proportions of patients with viral suppression and viral rebound were 76.1% and 21.0% respectively. Being diagnosed at WHO stage I [aOR = 11.40, 95% CI (3.54–36.74), *p* < 0.0001], having good adherence to ART [aOR = 5.09, 95% CI (2.67–9.73), *p* < 0.0001], taking Nevirapine-based regimen [aOR = 4.66, 95% CI (1.20–18.04), *p* = *0*.0260] and increasing duration of treatment (*p* < 0.0001) were independently associated with higher odds of viral suppression. However, being diagnosed at WHO stage II (aOR = 7.39, 95% CI 2.67–20.51; *p* < 0.0001) and stage III (aOR = 8.62, 95% CI 3.16–23.50; *p* < 0.0001), having poor adherence (aOR = 175.48, 95% CI 44.30–695.07; *p* < 0.0001), recording baseline suppression value of 20–49 copies/mL (aOR = 6.43, 95% CI 2.72–15.17; *p* < 0.0001) and being treated with Zidovudine/Lamivudine/Efavirenz (aOR = 6.49, 95% CI 1.85–22.79; *p* = 0.004) and Zidovudine/Lamivudine/Nevirapine (aOR = 18.68, 95% CI 1.58–220.90; *p* = *0*.02) were independently associated with higher odds of viral rebound.

**Conclusion:**

Approximately 76% viral suppression rate among HIV patients on ART in Kumasi falls below the WHO 95% target by the year 2030. Choice of ART combination, drug adherence, WHO clinical staging and baseline viral load are factors associated with suppression or rebound. These clinical characteristics of HIV patients must be monitored concurrently with the viral load.

## Introduction

Human immunodeficiency virus/acquired immune deficiency syndrome (HIV/AIDS) is the leading cause of death and morbidity in the world [1]. In 2015, 38.8 million people were living with HIV and over a million deaths were attributed to HIV [2]. Sub-Saharan Africa is the most affected region with more than two-thirds (75%) of new HIV infections and 75% global HIV/AIDS deaths [2, 3]. Ghana has HIV prevalence of 1.7%, affecting 334,713 people and accounting for over fourteen thousand annual deaths [4]. The Ahafo region (2.66%) and the Lower Manya Krobo District (5.56%) are the region and district with highest prevalence respectively, with the Ashanti region being the fifth HIV prevalent region (1.9%) in Ghana [4].

In response to the global HIV/AIDS mortality and morbidity, the Joint United Nations Programme on HIV/AIDS (UNAIDS) launched the “90–90–90” targets in 2014, to help eradicate HIV as part of Sustainable Development Goals (SDGs) [5]. By 2020, it was expected that 90% of people who are infected with HIV should know their status through testing, of whom 90% individuals who know their status should be put on antiretroviral therapy (ART) and 90% of those on medication should achieve viral suppression. However, in respect to attaining the “90–90–90” target, global assessment has shown that, of all people living with HIV, 79% know their status, 62% are assessing antiretroviral therapy and 53% has achieved viral suppression [6]. In Western and Central Africa, 64% of People living with HIV (PLWH) know their status, 51% of HIV positive individuals are accessing antiretroviral therapy and only 39% experience suppression [6]. In Ghana, 57% know their status of which 34% are on antiretroviral therapy [6]. However, limited data is available on those who experience suppression and rebound.

The adopted ART regimen in Ghana includes first line, second line and third line ART regimens. First line ART regimen include; Tenofovir + Lamivudine (or Emtricitabine) + Efavirenz second alternate as Tenofovir + Lamivudine (or Emtricitabine) + Nevirapine. The second line regimen is used when there is evidence of treatment failure with the first line regimen. This is confirmed by viral load monitoring. Second line ART regimen include; Zidovudine + Lamivudine (or Emtricitabine) + Lopinavir/r (or Atazanavir/r) with second alternate as Tenofovir + Lamivudine (or Emtricitabine) + Lopinavir/r (or Atazanavir/r). A third line therapy is recommended for those who have failed for second line treatment. Baseline investigation include laboratory testing for immunological function (CD4 + count) or patient’s viral load. Third line ART regimen includes; Darunavir/r + Dolutegravir or Raltegravir ± 1 or 2 NRTI with second alternate as DRV/r + 2NRTIs ± NNRTI [6].

The key focus on the fight against HIV/AIDS has been on the sustained antiretroviral therapy (ART), which has increased the success of viral suppression and continuous reduction in HIV/AIDS-related death [7]. Epidemiological studies have reported high rate of viral suppression among patients on first line ART, suggesting the hope of eradicating HIV if a streamlined care is utilized [8]. However, some people who achieve viral suppression are unable to maintain the viral load level and experience viral rebound [9].

Viral rebound increases vulnerability to other illnesses, treatment failure, ART resistance [10], and the potential for HIV transmission [11]. Again, viral rebound pose risk of increased HIV morbidity and mortality thereby hampering achieving UNAIDS agenda 95–95–95 targets by 2030. Although viral load uptake is common in Ghana, viral rebound following viral suppression has not received much attention. It is important to monitor these aspects of HIV/AIDS care to sustain the success achieved so far in the fight against HIV/AIDS.

Studies from other countries have related HIV viral suppression and viral rebound to numerous factors including adherence [11, 12], active tuberculosis [13], type of Antiretroviral therapy (ART) regimen [11, 14] and the socioeconomic status of the patient [15, 16]. To our knowledge, no study has evaluated the viral rebound and its associated factors in Ghana. For the first time, we evaluated viral suppression and viral rebound and their associated factors among HIV patients on antiretroviral therapy (ART) at the Komfo Anokye Teaching Hospital in Ghana.

## Materials and methods

### Study design and site

This hospital-based retrospective study was conducted at the Komfo Anokye Teaching Hospital (KATH). Participants’ data were retrieved from hospital archives and patients’ folders. KATH is the second major tertiary Hospital in Ghana. The facility has over 1000 bed capacity and serves as a referral center for other hospitals in the middle and northern belts of Ghana.

### Study population and sampling

**Fig. 1:**
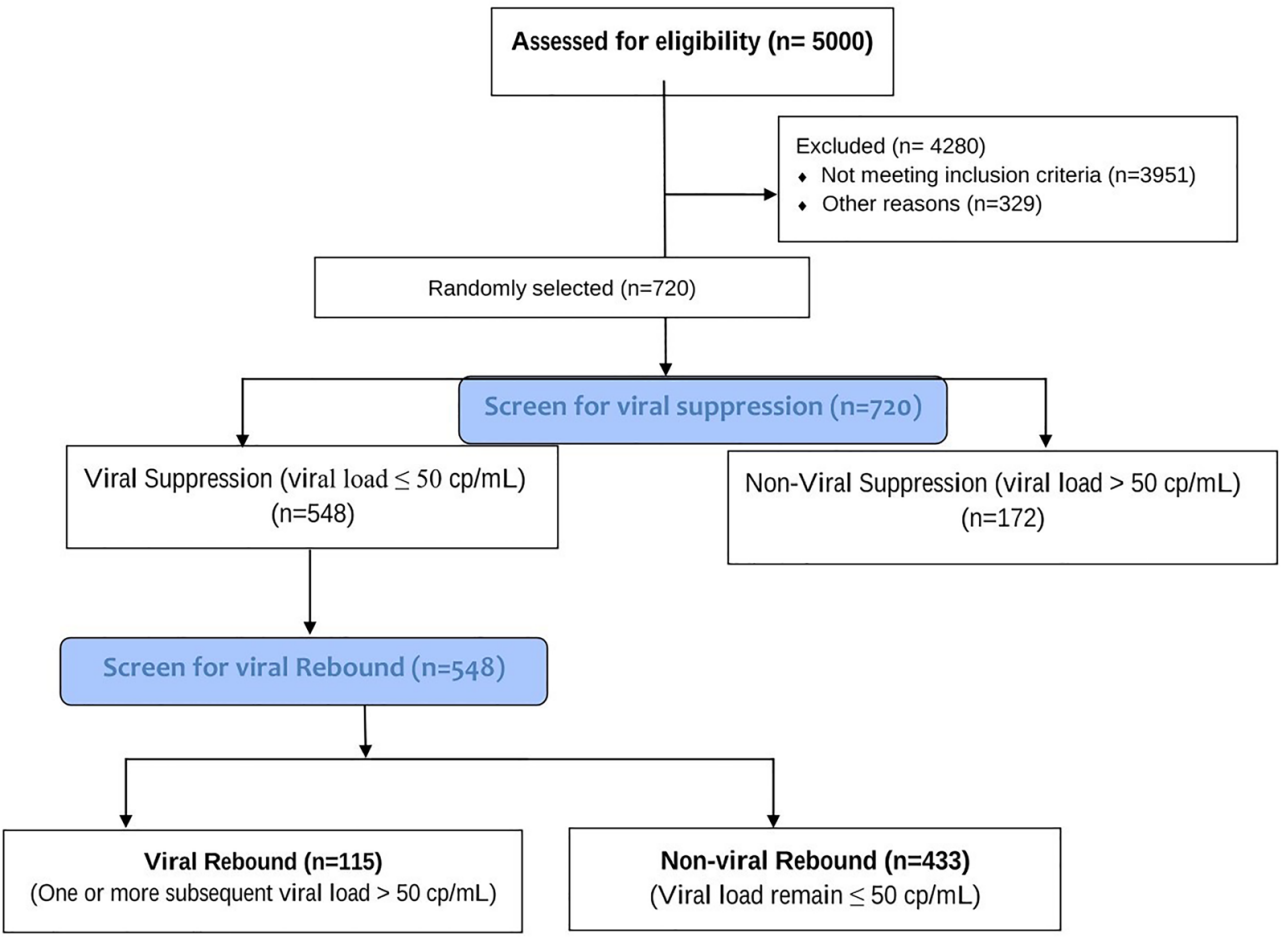
Consort flow chart

The study population included registered HIV patients receiving ART at KATH from 2016 to 2020. Records of a total number of 5000 HIV patients over a five-year period were reviewed. Of the 5000 patients, 4280 were excluded for not meeting inclusion criteria. Finally, 720 participants met the inclusion criteria and were included in the study. Of the 720 participants, 548 had achieved viral suppression and 172 did not achieve viral suppression. Of the 548 participants that achieved viral suppression, 115 experienced viral rebound and the remaining 433 maintained viral suppression (Fig. 1).

### Sample size calculation

The sample size was calculated using the online Calculator.net sample size calculator (https://www.calculator.net/sample-size-calculator.html). Where total population of 5000 registered HIV patients accessing the ART clinics of the three study sites from 2016 to 2020 with 95% confidence level, 50% response distribution, and 5% margin of error were employed. Substituting these values, the minimum sample size required in this study was 357. To increase statistical power, a total of 720 HIV patients were included in the study.

### Inclusion/exclusion criteria

All HIV-infected individuals 18 years and above, on ART for at least 6 months and with at least one valid report on viral load test were included in the study. All HIV-infected individuals below 18 years of age, ART naïve or on ART and without viral load results were excluded.

### Ethics consideration and consent

Ethical approval was sought from the Committee on Human Research, Publication and Ethics, School of Medical Sciences, Kwame Nkrumah University of Science and Technology (CHRPE/SMS/KNUST: CHEPE/AP/238/20). A thorough explanation of the study protocol and assurance of anonymity was made to the subjects. Written informed consent was also sought from participants and healthcare management before data and sample collection. All methods were carried out in accordance with relevant guidelines and regulations.

### Collection of sociodemographic and clinical data

Medical files of study participants were inspected and information on age, gender, religion, level of education, occupation and marital status, history of co-infections, opportunistic infections, type of HIV infection, ART regimens, adherence to treatment, and WHO stages of HIV/AIDS abstracted. The date and time patients were diagnosed and started ART were recorded. The date and results of the first viral load test (6 months after diagnosis) was taken as the baseline viral load. Date and results of subsequent viral load tests (12 months, 24 months, 36 months and 48 months) were recorded.

### Definition of viral suppression, viral rebound, adherence to and durability of viral suppression

Viral suppression was defined as recording at least one viral load less than 50 copies/mL after commencement of treatment [17–20] whilst viral rebound was defined as recording at least one viral load ≥ 50 copies/mL after being suppressed [17, 18]. Respective viral loads were taken 6 months apart per treatment guidelines and doctors reviews. Adherence was defined by using pill counts. In pill count, patients bring back the actual pill containers to the HIV clinic in order to retrospectively assess the number of pills that remain in the patients’ bottles. Patients were classified as good adherence if they do not miss pills in their history, fair adherence if they sometimes miss 1 or two pills and poor if they often miss more than two pills [21, 22]. Durability of viral suppression was defined as the length of time that patients on ART with viral load suppression will be able to maintain prior to viral rebound [23].

### Statistical analysis

Collected data obtained were entered, coded, edited, and cleaned in Microsoft Excel 2016. All statistical analyses were performed using the Statistical Package for Social Sciences (SPSS) Version 26.0 (Chicago IL, USA) and GraphPad Prism version 8.0 (GraphPad Software, San Diego California USA, www.graphpad.com). Data collected in this study were categorical data and were therefore presented as frequencies and percentages. A bar chart was used to illustrate the prevalence of viral suppression among study participants. Univariate logistic regression analysis was performed to screen for potential clinical and socio-demographic characteristics associated with viral suppression and viral rebound. Multivariate logistic regression model was used to determine independent predictors of viral suppression and viral rebound among HIV patients on treatment. p-values less than 0.05 (*p* < 0.05) were considered statistically significant.

## Results

### Sociodemographic and clinical characteristics of study participants

A total of 720 participants were included in the study. About one-third of the study participants were 30–39 years (31.8%) or 40–49 years (32.5%). The majority of the patients in this study were females (74.4%), married (44.6%), have had junior high school (33.2%) or primary school education (22.1%), were working in the informal sector (73.2%) or were Christians (85.4%) (Table 1).

**Table 1:** Sociodemographic characteristics of study participants

Variable	Number of participants (n = 720)	Percentage (%)
Age category (years)		
18–29	84	11.7
30–39	229	31.8
40–49	234	32.5
50–59	127	17.6
60 and above	46	6.4
Gender		
Male	184	25.6
Female	536	74.4
Marital status		
Married	321	44.6
Single	197	27.4
Cohabiting	34	4.7
Widow/widower	75	10.4
Divorced	93	12.9
Educational level		
No formal education	99	13.8
Primary school	159	22.1
Junior high school	239	33.2
Senior high school	152	21.1
Tertiary education	71	9.9
Occupation		
Unemployed	126	17.5
Informal	527	73.2
Formal	67	9.3
Religion		
Christian	615	85.4
Muslim	94	13.1
Traditionalist	1	0.1
Other (non-affiliated)	10	1.4

Other = any other religion apart from Christian, Muslim or Traditionalist

Majority of the patients were infected with HIV-1 (96.0%), were diagnosed at WHO stage I (38.8%) or stage III (35.8%) or had no past antiretroviral (ARV) experience (97.9%). More than half (51.8%) of the patients had good adherence to ART. A few participants had other co-morbidities (diabetes mellitus, asthma, hypertension and tuberculosis) (11.1%), opportunistic infections (10.8%), or had ever stopped or changed ARV (7.6%). The majority of the patients were on the Efavirenz-based regimen (79.7%) and a few participants were on Lopinavir (PI)-based regimen (5.0%). Again, more than half of the participants were taking TDF/3TC/EFV (69.6%) as the specific ARV combination (Table 2).

**Table 2:** Clinical characteristics of study participants

Variable	Number of participants (n = 720)	Percentage (%)
HIV type		
Type 1	691	96.0
Type 2	5	0.7
Type 1 and 2	24	3.3
WHO stage of HIV		
Stage I	243	38.8
Stage II	193	26.8
Stage III	258	35.8
Stage IV	26	3.6
Past ARV experience		
No	705	97.9
Yes	15	2.1
Adherence to ART		
Good	373	51.8
Fair	254	35.3
Poor	93	12.9
Presence of other conditions		
No	640	88.9
Yes	80	11.1
Presence of opportunistic infection (s)		
No	642	89.2
Yes	78	10.8
Ever stopped or changed ARV		
No	685	92.4
Yes	55	7.6
ARV regimen		
Efavirenz-based	574	79.7
Nevirapine-based	63	8.8
Lopinavir (PI)-based	36	5.0
Integrase-based	47	6.5
ARV combinations		
TDF/3TC/EFV	501	69.6
TDF/FTC/EFV	5	0.7
AZT/3TC/EFV	44	6.1
ABC/3TC/EFV	7	1.0
TDF/3TC/NVP	34	4.7
TDF/FTC/NVP	2	0.3
AZT/3TC/NVP	44	6.1
TDF/3TC/LPV/r	29	4.0
TDF/FTC/LPV/r	4	0.6
AZT/3TC/LPV/r	3	0.4
TDF/3TC/DTG	47	6.5

*HIV* human immunodeficiency virus, *WHO* World Health Organization, *ARV* antiretroviral, *ART* antiretroviral therapy, *TDF* tenofovir, *3TC* lamivudine, *EFV* efavirenz, *ABC* abacavir, *FTC* emtricitabine, *NVP* nevirapine, *AZT* zidovudine, *LPV/r* lopinavir/ritonavir, *DTG* dolutegravir

### Proportion of HIV viral suppression and rebound among HIV patients of on treatment

**Fig. 2:**
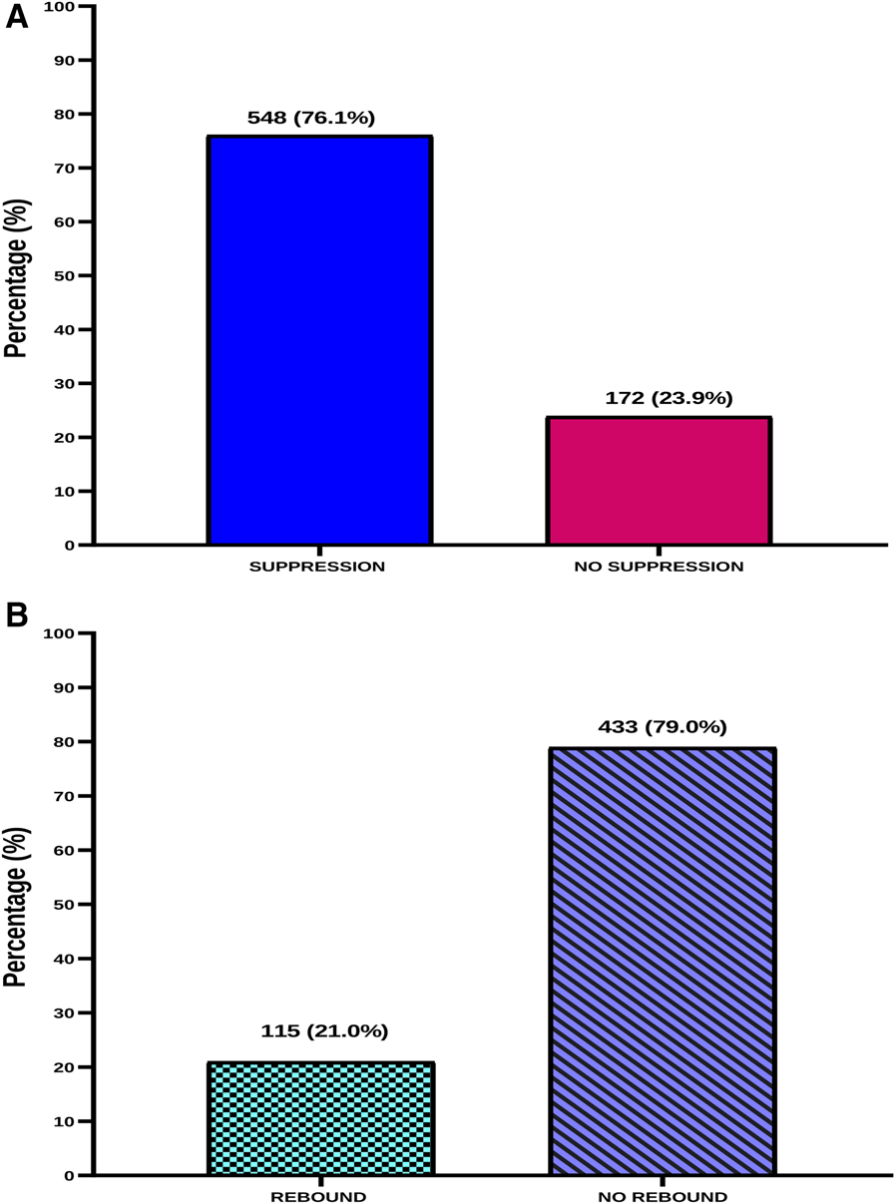
Viral suppression (**A**) and rebound (**B**) among HIV patients on treatment

Of 720 participants that were analyzed in this study, the proportion of participants that achieved viral suppression (viral load < 50 copies/mL) was 548 representing 76.1% (Fig. 2A). Of 548 participants who achieved viral suppression, 21.0% experienced viral rebound (Fig. 2B).

### Factors associated with viral suppression among HIV patients on treatment

In a univariate logistic regression model, being diagnosed in WHO stage I, having good and fair adherence to ART, taking TDF/3TC/EFV, AZT/3TC/EFV or AZT/3TC/NVP and increasing duration of treatment were significantly associated with higher odds of attaining viral suppression compared to WHO stage IV, bad adherence to ART, taking Integrase based-regimen, and treatment for less than one year respectively.

After adjusting for possible cofounders in a multivariate logistic regression model, being diagnosed at WHO stage I [aOR = 11.40, 95% CI (3.54–36.74), *p* < 0.0001], having good adherence to ART [aOR = 5.09, 95% CI (2.67–9.73), *p* < 0.0001], being treated with TDF/3TC/EFV [aOR = 3.00, 95% CI (1.15–7.78), *p* = *0*.0240], AZT/3TC/EFV [aOR = 6.83, 95% CI (1.83–25.45), *p* = *0*.0040] or AZT/3TC/NVP [aOR = 5.16, 95% CI (1.33–19.94), *p* = *0*.0170] and increasing duration of treatment were independently associated with increased odds of viral suppression. However, ever stopping or changing ARV [aOR = 0.20, 95% CI (0.05–0.70), *p* = *0*.0190] was significantly associated with lower odds of viral suppression (Table 3).

**Table 3:** Predictors of viral suppression among HIV patients on ART

Variable	Viral suppression (n = 548)	cOR (95% CI)	*p*-value	aOR (95% CI)	*p*-value
WHO stage of HIV					
Stage I	224 (40.9)	7.37 (2.94–18.46)	**< 0.0001**	11.40 (3.54–36.74)	**< 0.0001**
Stage II	135 (24.6)	1.46 (0.62–3.40)	0.3860	2.13 (0.71–6.42)	0.1800
Stage III	173 (31.6)	1.27 (0.55–2.92)	0.5710	1.32 (0.45–3.89)	0.6100
Stage IV	16 (2.9)	Ref. (1)	–	Ref. (1)	–
Adherence to ART					
Good	326 (59.5)	6.79 (4.08–11.29)	**< 0.0001**	5.09 (2.67–9.73)	**< 0.0001**
Fair	175 (31.9)	2.17 (1.33–3.52)	**0.0020**	1.78 (0.95–3.35)	0.0720
Poor	47 (8.6)	Ref. (1)	–	Ref. (1)	–
Ever stopped or changed ARV					
No	499 (91.1)	Ref. (1)	–	Ref. (1)	–
Yes	49 (8.9)	0.368 (0.16–0.88)	**0.0240**	0.20 (0.50–0.70)	**0.0190**
Common ARV combinations^a^					
TDF/3TC/EFV	381 (69.5)	3.61 (1.96–6.63)	**< 0.0001**	3.00 (1.15–7.78)	**0.0240**
AZT/3TC/EFV	37 (6.8)	6.01 (2.23–16.17)	**< 0.0001**	6.83 (1.83–25.45)	**0.0040**
TDF/3TC/NVP	28 (5.1)	5.30 (1.85–15.18)	**0.0020**	2.57 (0.61–10.76)	0.1960
AZT/3TC/NVP	39 (4.6)	8.86 (2.97–26.45)	**< 0.0001**	5.16 (1.33–19.94)	**0.0170**
TDF/3TC/LPV/r	25 (4.6)	7.10 (2.14–23.60)	**0.0010**	3.58 (0.74–17.40)	0.1140
TDF/3TC/DTG	22 (4.0)	Ref. (1)		Ref. (1)	–
Duration of ARV treatment (years)					
< 1	5 (0.9)	Ref. (1)		Ref. (1)	–
1	105 (19.2)	5.81 (2.14–15.74)	**0.0010**	6.52 (2.05–20.69)	**0.0010**
2	204 (37.2)	31.20 (11.21–86.84)	**< 0.0001**	38.04 (11.52–125.61)	**< 0.0001**
3	207 (37.8)	63.32 (21.56–185.95)	**< 0.0001**	79.93 (23.08–276.78)	**< 0.0001**
4	27 (4.9)	140.40 (15.35–1284.39)	**< 0.0001**	179.82 (17.21–1879.35)	**< 0.0001**

bold values indicate the *p*-value is statistically significant

*HIV* human immunodeficiency virus, *WHO* World Health Organization, *ARV* antiretroviral, *ART* antiretroviral therapy, *TDF* tenofovir, *3TC* lamivudine, *EFV* efavirenz, *ABC*; abacavir, *FTC* emtricitabine, *NVP* nevirapine, *AZT* zidovudine, *LPV/r* lopinavir/ritonavir, *DTG* dolutegravir

^a^Variables with missing values

### Factors associated with viral rebound among HIV patients on treatment

In a multivariate logistic regression model, being diagnosed at WHO stage II (aOR = 7.39, 95% CI 2.67–20.51; *p* < 0.0001) and WHO stage III (aOR = 8.62, 95% CI 3.16–23.50; *p* < 0.0001), having fair (aOR = 8.71, 95% CI 3.96–19.18; *p* < 0.0001), or poor adherence (aOR = 175.48, 95% CI 44.30–695.07; *p* < 0.0001), recording a baseline viral suppression value of 20–49 copies/mL (aOR = 6.43, 95% CI 2.72–15.17; *p* < 0.0001), being treated with AZT/3TC/EFV (aOR = 6.49, 95% CI 1.85–22.79; *p* = *0*.0040) or AZT/3TC/NVP (aOR = 18.68, 95% CI 1.58–220.90; *p* = *0*.02), obtaining durability of ARVs viral suppression for up to 24 months (aOR = 4.63, 95% CI (1.34–24.48); *p* < 0.0001) were independently associated with higher odds of viral rebound.

However, being diagnosed in WHO stage IV, having other conditions or opportunistic infections, recording baseline suppression value less than 20 copies/mL, ever stopped or changed ARV were not significantly associated with HIV viral rebound (Table 4).

**Table 4:** Factors associated with viral rebound among HIV Patients on treatment

Variable	Viral rebound (n = 115)	cOR (95% CI)	p value	aOR (95% CI)	*p* value
WHO stage of HIV					
Stage I	15 (13.0)	Ref. (1)	–	Ref. (1)	–
Stage II	37 (32.2)	5.26 (2.76–10.04)	**< 0.0001**	7.39 (2.67–20.51)	**< 0.0001**
Stage III	60 (52.2)	7.40 (4.02–13.62)	**< 0.0001**	8.62 (3.16–23.50)	**< 0.0001**
Stage IV	3 (2.6)	3.22 (0.83–12.532)	0.092	4.01 (0.47–34.06)	0.2040
Adherence to ART					
Good	20 (17.4)	Ref. (1)	–	Ref. (1)	–
Fair	59 (51.3)	7.78 (4.49–13.49)	**< 0.0001**	8.71 (3.96–19.18)	**< 0.0001**
Poor	36 (31.3)	50.07 (22.21–112.87)	**< 0.0001**	175.48 (44.30–695.07)	**< 0.0001**
Presence of other condition (s)					
No	94 (81.7)	Ref. (1)	–	Ref. (1)	–
Yes	21 (18.3)	1.84 (1.05–3.22)	**0.034**	1.99 (0.77–5.14)	0.1550
Presence of opportunistic infection (s)					
No	94 (81.7)	Ref. (1)	–	Ref. (1)	–
Yes	21 (18.3)	2.62 (1.46–4.72)	**0.0010**	1.23 (0.39–3.90)	0.7210
Baseline suppression category					
Target not detected	26 (22.8)	Ref. (1)	–	Ref. (1)	–
< 20	23 (20.2)	1.69 (0.92–3.09)	0.0880	1.62 (0.65–3.99)	0.2990
20–49	65 (57.0)	8.25 (4.87–13.98)	**< 0.0001**	6.43 (2.72–15.17)	**< 0.0001**
Ever stopped or changed ARV					
No	97 (84.3)	Ref. (1)	–	Ref. (1)	–
Yes	18 (15.7)	2.41 (1.29–4.48)	**0.0060**	1.61 (0.45–5.73)	0.4600
Common ARV combinations					
TDF/3TC/EFV	46 (40.0)	Ref. (1)	–	Ref. (1)	–
AZT/3TC/EFV	22 (19.1)	10.68 (5.17–22.06)	**< 0.0001**	6.49 (1.85–22.79)	**0.0040**
TDF/3TC/NVP	8 (7.0)	2.91 (1.21–6.99)	**0.0170**	3.48 (0.53–22.87)	0.1950
AZT/3TC/NVP	24 (20.9)	11.65 (5.70–23.82)	**< 0.0001**	18.68 (1.58–220.90)	**0.0200**
TDF/3TC/LPV/r	8 (7.0)	3.43 (1.40–8.39)	**0.0070**	2.26 (0.56–9.19)	0.2520
TDF/3TC/DTG	1 (0.9)	0.35 (0.05–2.64)	0.3060	0.21 (0.02–2.26)	0.1990
Durability of ARV (months)					
0–6	36 (31.3)	19.41 (4.45–84.76)	**< 0.0001**	8.87 (2.92–31.36)	**< 0.0001**
7–12	34 (29.6)	8.74 (2.02–37.73)	**0.0040**	6.70 (2.23–22.91)	**< 0.0001**
13–24	34 (29.6)	7.36 (1.71–31.73)	**0.0070**	4.63 (1.34–24.48)	**< 0.0001**
25–36	9 (7.8)	2.72 (0.57–13.05)	0.2110	3.68 (0.75–13.21)	0.0940
37–48	2 (1.7)	Ref. (1)	–	Ref. (1)	–

Adjusted for age and gender

bold values indicate the *p*-value is statistically significant

*HIV* human immunodeficiency virus, *WHO* World Health Organization, *ARV* antiretroviral, *ART* antiretroviral therapy, *TDF* tenofovir, *3TC* lamivudine, *EFV* efavirenz, *ABC* abacavir, *FTC* emtricitabine, *NVP* nevirapine, *AZT* zidovudine, *LPV/r* lopinavir/ritonavir, *DTG* dolutegravir, *cOR* crude odd ratio, *aOR* adjusted odd ratio, *CI* confidence interval

## Discussion

Human immunodeficiency virus/acquired immune deficiency syndrome (HIV/AIDS) is a leading cause of death worldwide. The fight against HIV/AIDS is hinged on initiation and adherence to an effective Antiretroviral Therapy (ART), and this has increased the attainment of viral suppression and reduction in HIV/AIDS-related death. Unfortunately, some people who achieve viral suppression are unable to maintain it and experience viral rebound, increasing their risk of treatment failure and the potential for transmission.

The United Nation's principal goal for eradicating HIV/AIDS by 2030 involves expanding access to and coverage of ART across the globe and viral suppression [24–26]. However, global assessment has shown that only around half of the world’s HIV patients on ART are currently virally suppressed [25, 27]. In the present study, the proportion of patients who attained viral suppression was 76.1%. Consistent but quite higher than our study finding, Lebelonyane et al. [28] and Koss et al. [29], reported viral suppression among 82% of PLWH in Botswana and 80.7% among those in Uganda [28, 29]. Our study finding is higher compared to studies by Lokpo et al. [30] and Maina et al. [11], who reported 69% and 59% viral suppression rate among patients in the Volta region of Ghana and Kenya respectively [11, 30]. Again, viral suppression rate observed in our study is much higher compared to 24% in Sierra Leone and 41% in Senegal [25]. Although the 90% UNAIDS target for viral suppression is much higher than what was found in this study, our results provide a glimpse into the country’s progression towards the UNAIDS 90–90–90 agenda.

We explored the factors associated with viral suppression among patients on ART. We observed that being diagnosed with WHO stage I, having good adherence to ART, being on a Nevirapine-based regimen and increasing duration of treatment with ARV were independently associated with higher chances of viral suppression. However, stopping or changing ARVs was associated with lower chances of viral suppression. Our findings are comparable to those observed by Maina et al. [11], who reported WHO stage I of HIV infection and good adherence to ART were associated with an increased likelihood of viral suppression [11]. Moreover, a study by O’Connor et al. [23], found good ART adherence, and being diagnosed in WHO stage I were associated with viral suppression. The findings for WHO stage I being associated with high chances of viral suppression could be attributed to the initial infection stage of the virus and less downregulation of the immune system at this stage, making the body’s natural immune system still responsive to infection.

In our study, increasing duration of treatment was associated with higher chances of viral suppression, consistent with a study by Kiselinova et al. [31] in the United Kingdom. This supports evidence from clinical studies that suggests prolonged ART use can reduce viral load below the limit of detection for a long-term period [32]. Moreover, stopping or changing ARVs was associated with lower chances of viral suppression. A similar finding was reported by Martínez et al. [33] and Maman et al. [34], who observed that switching HIV patients from a protease inhibitor to nevirapine, efavirenz, or abacavir resulted in a higher rate of virologic failure [33, 34]. Hence, the type of ARV regimen may influence viral suppression and the risk of viral rebound.

Viral rebound increases vulnerability to illness, treatment failure, ART resistance, and the potential for HIV transmission [35, 36]. In the current study, viral rebound occurred in 21.0% of our patients. Our study finding is comparable but much higher than findings of Craw et al. [37], who reported 7.5% viral rebound rate among American HIV patients who had achieved viral suppression [37]. In Kenya, among PLWH, viral rebound was 41% [11]. This implies that a considerable number of HIV patients who achieve viral suppression are unable to remain suppressed and therefore experience rebound episodes. This may contribute to some of the reasons why Ghana could not achieve the UNAIDS agenda 90–90–90 target in 2020.

We evaluated a number of putative factors associated with viral rebound. In the current study, we observed that patients diagnosed at WHO stage II and stage III had higher chances of viral rebound compared to those diagnosed at WHO stage I. Our study finding is in harmony with a study by Maina et al. [11], who reported that being diagnosed at WHO stage II is associated with higher viral rebound [11]. These findings suggests that patients at WHO stage II and above have downregulated immune system with associated opportunistic infections and comorbidities, increasing their venerability to viral rebound [38].

Notably, we observed that poor adherence was associated with higher viral rebound. Consistent with our study findings, Bulage et al. [39] reported higher viral rebound among HIV patients who experience rebound episodes. Among Kenyan HIV patients, Maina et al. [11], reported that poor ART adherence is associated with viral rebound [11]. The agreement between the current and previous studies may explain why majority of non-adherent HIV patients are vulnerable to illness, experience treatment failure and ART resistance [35, 36]. Furthermore, we observed that patients who recorded a baseline viral suppression value of 20–49 copies/mL had higher chances of viral rebound compared to those who had target not detected. Consistent with our study finding, Palmer et al. [40], reported that baseline suppression count is associated with viral rebound [40]. Among Chinese HIV patients, Li et al. [41], reported similar association of baseline suppression value and viral rebound [41]. This finding is suggestive that patients with the lowest levels of viral load to the target not detected are more stable and unlikely to experience viral rebound.

Moreover, we observed that patients on Zidovudine based-regimen (AZT/3TC/EFV and AZT/3TC/NVP) had higher chances of viral rebound compared to patients on Tenofovir based-regimen (TDF/3TC/EFV). This is in line with a study by Mania et al. [11], who observed that AZT/ 3TC/NVP regimen was associated with high viral rebound. NNRTI- and NRTI-based regimens have increased risk of resistance within the first six months of treatment [42].

Our study provides useful findings to guide HIV clinicians and policy makers in the fight against the infection. However, it is limited by the fact that it was a retrospective study and single-centered. We relied on available hospital data, especially patients’ viral load results, which posed a challenge to completeness of data. Moreover, some variable such as duration of treatment had larger confidence intervals for predicting viral suppression and rebound indicating less power and therefore a larger study is needed to generate enough evidence of the estimates.

## Conclusion

The viral suppression rate among HIV patients on ART in Kumasi (76%) do not meet the WHO target (90%). Good adherence to ART, being on Nevirapine-based regimen and increasing duration of treatment with ARV independently leads to viral suppression. However, being diagnosed at WHO stage II and stage III, having bad to fair adherence, recording baseline suppression value of 20–49 copies/ml and being treated with AZT/3TC/EFV and AZT/3TC/NVP leads to viral rebound. These clinical characteristics of HIV patients, associated with viral suppression and viral rebound must be monitored concurrently with the viral load.

## Data Availability

All data generated or analyzed during this study are included in this article and its additional information files data and can be requested from corresponding author.

## Abbreviations

HIV: Human immunodeficiency virus
WHO: World Health Organization
ARV: Antiretroviral
ART: Antiretroviral therapy
TDF: Tenofovir
3TC: Lamivudine
EFV: Efavirenz
ABC: Abacavir
FTC: Emtricitabine
NVP: Nevirapine
AZT: Zidovudine
LPV/r: Lopinavir/ritonavir
DTG: Dolutegravir
CHRPE: Committee on Human Research Publication and Ethics
KATH: Komfo Anokye Teaching Hospital
KNUST: Kwame Nkrumah University of Science and Technology
